# Acute cerebellitis following COVID-19 infection associated with autoantibodies to glutamate receptors: a case report

**DOI:** 10.1007/s13365-023-01183-7

**Published:** 2023-11-06

**Authors:** Takuya Watanabe, Yuki Kakinuma, Keiko Watanabe, Ryuta Kinno

**Affiliations:** Division of Neurology, Department of Internal Medicine, Showa University Northern Yokohama Hospital, 35-1 Chigasaki-chuo Tsuzuki-ku, Yokohama, Kanagawa 224-8503 Japan

**Keywords:** Acute cerebellitis, Autoantibody, Cerebrospinal fluid, Glutamate receptor, Post-COVID-19 infection

## Abstract

While COVID-19 infection by the SARS-CoV-2 virus was initially identified as a respiratory disease, mounting evidence suggests its association with various neurological issues as well. Notably, COVID-19 has been linked to acute cerebellitis (AC) and post-infectious cerebellar ataxia. The precise underlying mechanisms behind these neurological effects remain unclear. Our case report describes AC following COVID-19 infection, associated with autoantibodies to glutamate receptors (GluRs), hinting at immunological involvement. The case is a 56-year-old woman who experienced fever and fatigue due to COVID-19 infection. About 2 weeks after these symptoms improved, she showed cerebellar symptoms such as ocular overshoot and ataxia when presenting to our hospital. Her cerebrospinal fluid (CSF) findings were normal. Brain MRI revealed cerebellar abnormalities. Treatment with methylprednisolone led to symptom improvement. Later tests of CSF yielded positive results for autoantibodies to GluRs. Our findings suggest a possible immune-mediated mechanism in the onset of AC following COVID-19 infection. Clinicians should consider the possibility of immunological pathogenesis when diagnosing cerebellar symptoms after COVID-19 infection.

## Introduction

While COVID-19 infection was initially identified as a respiratory disease, evidence for various neurological problems in affected patients has been accumulating (Ellul et al. 2020). Previous studies have revealed a distinct association between COVID-19 and acute cerebellitis (AC), along with post-infectious cerebellar ataxias (Fadakar et al. 2020). However, the exact mechanisms responsible for these neurological effects are not fully understood. Previous research has suggested a possible association between AC and a specific type of autoantibody to the glutamate receptor (GluR), namely GluR δ2 (Kinno et al. 2013). Here we present a case of AC following COVID-19 infection. This case was also associated with autoantibodies to GluRs, including GluR δ2, suggesting the possible involvement of immunologic mechanisms.

## Case report

A 56-year-old woman experienced mild fever and fatigue (day 0), prompting her to seek medical attention at various medical facilities. Her nasopharyngeal swab polymerase chain reaction (PCR) for COVID-19 was positive. Fever and fatigue disappeared a few days after onset. About 2 weeks after these symptoms improved, she experienced difficulty with arm and hand mobility, speech articulation, and maintaining balance while standing. She consulted our department three days after the onset of these neurological symptoms (on day 22). On admission, her body temperature was 36.5 °C; other vital signs were normal. Despite an otherwise normal general exam, manifestations suggestive of cerebellar impairment were present. These signs included oculomotor overshoot, gaze-evoked nystagmus, and dysarthria with scanning speech. Further evaluation revealed pronounced ataxia involving both limbs and trunk, as well as intention tremor. The finger-to-nose and heel-to-knee tests revealed impaired coordination of limb movements. Muscle tone was hypotonic. Cranial nerve examination revealed no abnormalities. Muscle strength was normal and deep tendon reflexes were within normal limits. There were no sensory disturbances and no meningeal signs.

**Fig. 1:**
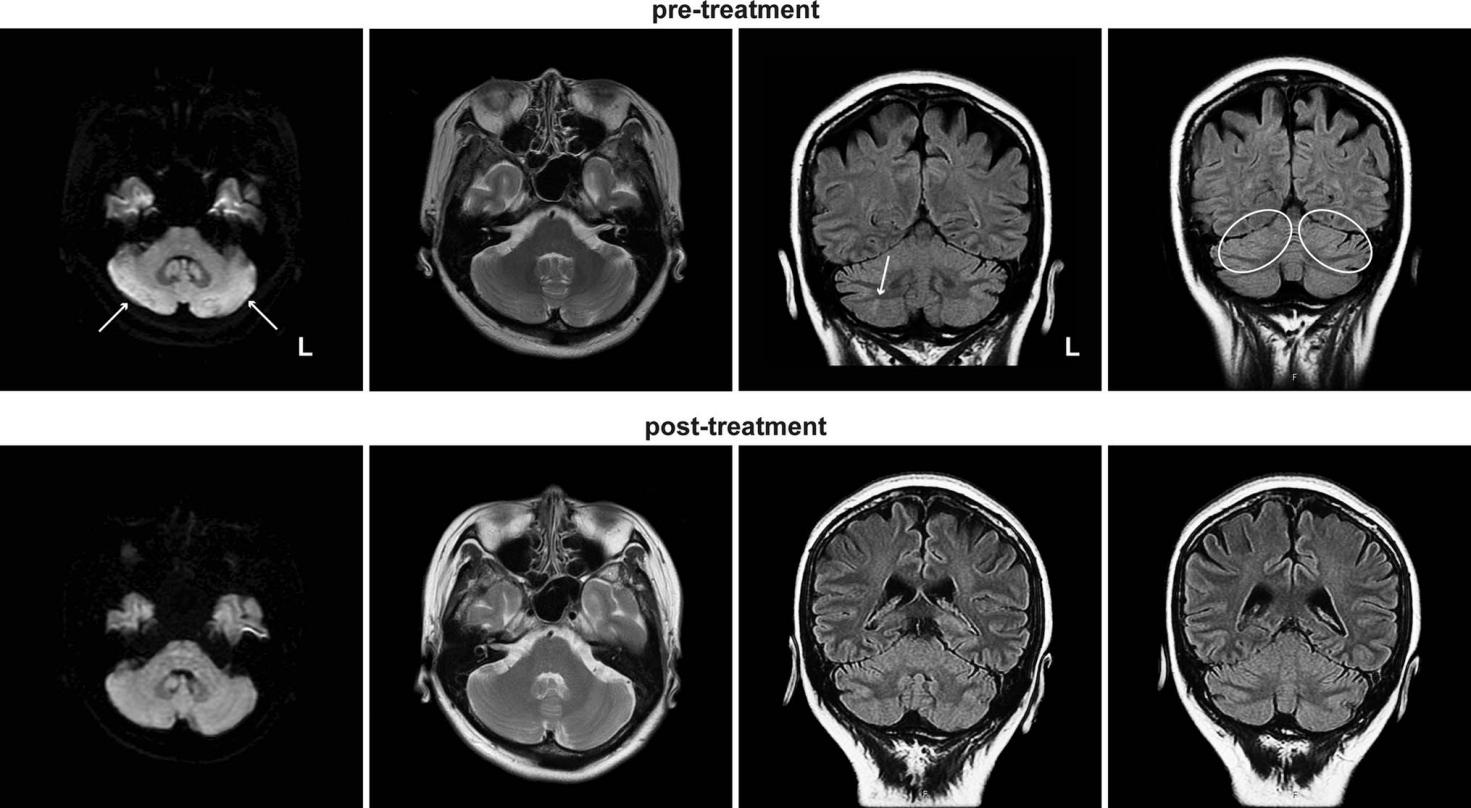
MRI findings of the patient. Axial DWI (left), axial T2-weighted image (middle left), and coronal FLAIR images (middle right and right) are shown. The hyperintensity in the cerebellum (arrows in DWI and FLAIR image) and edematous changes (circles in FLAIR image) was resolved after methylprednisolone treatments

Nasopharyngeal swab PCR for COVID-19 on admission was negative. Blood tests revealed a slightly elevated erythrocyte sedimentation rate (40 mm/h), while the white blood cell count remained within the normal range (6220 /μL with 69.5% neutrophils). Cerebrospinal fluid (CSF) analysis showed a normal cell count (2/μL), along with typical levels of glucose (61 mg/dL) and protein (35 mg/dL). The oligoclonal immunoglobulin G (IgG) band was absent. PCR for herpes simplex virus and varicella zoster virus deoxyribonucleic acid (DNA) in CSF were negative. A variety of tests were performed on serum samples, including those for antinuclear antibodies, anti-ds-DNA, SS-A, SS-B, anti-GM1 IgG antibody, anti-GQ1b IgG antibody, and paraneoplastic antibodies (anti-amphiphysin, anti-CV2, anti-Ma2/Ta, anti-Ri, anti-Yo, anti-Hu, anti-revoverin, anti-SOX1, anti-titin, anti-zic4, anti-GAD65, and anti-Tr antibodies); all yielded negative results. On day 27, a brain MRI showed hyperintensity in the cerebellum on diffusion-weighted imaging (DWI) (Fig. 1). The T2/fluid-attenuated inversion recovery (FLAIR) showed edematous changes with slightly increased signals compared with the adjacent tissue. A whole-body CT scan showed no abnormalities, eliminating the possibility of malignancy.

Subsequently, two courses of treatment with intravenous methylprednisolone (1 g/day) for 3 days were administered. After the second course, the patient’s cerebellar symptoms gradually improved, and the brain MRI abnormalities resolved (Fig. 1). On day 50, she was transferred to a specialized rehabilitation facility. Subsequent tests performed on the CSF (collected on day 22) yielded positive results for autoantibodies to GluRs (Table 1). Thus, we arrived at a diagnosis of AC associated with autoantibodies to GluRs.

**Table 1:** Optical density of antibody to GluR

	GluRε2-NT2	GluRε2-CT1	GluRΖ1-NT	GluRδ2-NT
CFS	0.797 (0.254 ± 0.075)	0.956 (0.331 ± 0.110)	0.907 (0.323 ± 0.091)	0.983 (0.378 ± 0.117)

Optical densities assessed by ELISA are shown (mean ± standard deviation for control data)

## Discussion

In our case, cerebellar symptoms manifested 2 to 3 weeks after COVID-19 infection, but CSF analysis were normal. MRI scans showed abnormalities consistent with cerebellitis. Administration of intravenous methylprednisolone successfully relieved the cerebellar symptoms. These clinical features resemble those documented in previous literature (Malayala et al. 2021). The authors suggested an immune-mediated mechanism underlying cerebellitis after COVID-19 infection, although no detectable antibodies were found in their case. In contrast, our case showed positive results for autoantibodies to several types of GluRs in the CSF (Table 1).

The hyperintensity in the cerebellum was prominent on DWI, but mild and localized on FLAIR (Fig. 1), similar to the previous case (Fadakar et al. 2020). The DWI-FLAIR mismatch is often observed in acute ischemic stroke (Thomalla et al. 2011). However, the MRI signal changes in our case were bilateral and reversible, similar to those in mild encephalitis/encephalopathy with reversible splenic lesion (MERS), rather than stroke. Its MRI abnormalities are more prominent on DWI than on FLAIR image and are reversible. Although the pathophysiological mechanism remains unclear, a previous case report on MERS with COVID-19 suggested that intramyelinic edema and inflammation might result from a spontaneous reversible autoimmune response, with complete resolution of clinical and radiographic abnormalities as seen in most cases (Kakadia et al. 2020). The presence of GluRs may link to this response.

GluRδ2 has been shown to be prominently expressed in cerebellar Purkinje cells and to play a central role in cerebellar functionality (Yuzaki 2004). In addition, previous literature has reported cases of AC associated with GluRδ2 (Kinno et al. 2013). Unlike GluRδ2, the expression of GluRε2 mRNA is restricted to the forebrain, including the cerebral cortex and limbic system postnatally. GluRε2 has been implicated in memory and learning processes (Tang et al. 1999). We surmised that the observed cerebellar symptoms were related to the autoantibody against GluRδ2. Our case serves as an indicator and provides potential evidence for an immune-mediated mechanism in the development of AC following COVID-19 infection.

This report is a single case report; thorough investigations involving larger patient cohorts and experimental studies are essential to elucidate the underlying mechanisms and potentially develop therapeutic strategies to manage neurologic complications in individuals affected by COVID-19. Nevertheless, the observations from this case have pertinent implications for clinicians who encounter cases of AC following COVID-19 infection. The differential diagnosis for AC includes considerations such as stroke, infectious meningoencephalitis, cerebellar tumors, acute disseminated encephalomyelitis, and posterior reversible encephalopathy syndrome. Careful efforts to rule out alternative etiologies are critical (Van Samkar et al. 2017). Clinicians should consider the possibility of immunological pathogenesis when diagnosing cerebellar symptoms after COVID-19 infection.

## Data Availability

The data that support the findings of this study are available from the corresponding author upon reasonable request.
